# Defining and classifying public health systems: a critical interpretive synthesis

**DOI:** 10.1186/s12961-020-00583-z

**Published:** 2020-06-16

**Authors:** Tamika Jarvis, Fran Scott, Fadi El-Jardali, Elizabeth Alvarez

**Affiliations:** 1 Department of Health Research Methods, Evidence and Impact (HEI), McMaster University, 1280 Main Street West, Hamilton, ON L8S 4K1 Canada; 2 Faculty of Health Sciences, American University of Beirut, Beirut, Lebanon

**Keywords:** Public health, Health systems, Population health, Health services

## Abstract

**Background:**

The introduction of the determinants of health has caused a shift towards understanding health from a holistic perspective as well as increased recognition of public health’s contributions to the health of the population. Several frameworks exist to conceptualise healthcare systems, highlighting the stark contrast of frameworks unique to public health systems. The objectives of this study were to define public health systems and assess differences between healthcare systems and public health systems within established health systems frameworks.

**Methods:**

A critical interpretive synthesis was conducted. Databases searched included EBSCOhost, OVID, Scholars Portal, Web of Science, Cochrane Library and Health Systems Evidence. Data extraction, coding and analysis followed a best-fit framework analysis method. Initial codes were based on a current leading health systems and policy classification scheme – health systems arrangements (governance, financial and delivery arrangements).

**Results:**

A total of 5933 unique documents were identified and 67 were included in the analysis. Definitions of public health and public health systems varied significantly as did their roles and functions across jurisdictions. Public health systems arrangements generally followed those of health systems, with the addition of partnerships (community and inter-sectoral) and communication playing a larger role in public health. A public health systems framework and conceptualisation of how public health currently fits within health systems are presented.

**Conclusions:**

Public health systems are unique and vital entities within health systems. In addition to examining how public health and public health systems have been defined within the literature, this review suggests that establishing the scope of public health is crucial to understanding its role within the larger health system and adds to the discourse around the relationship between public health, healthcare and population health. More broadly, this study addresses an important gap in understanding public health systems and provides conceptual and practical contributions as well as areas for future research.

## Background

Public health is generally understood to engage in population rather than in individual health activities and to undertake a population health approach recognising that genetic, behavioural and socio-economic factors (e.g. housing, social networks, education) influence health and well-being [1, 2]. The introduction of the determinants of health has caused a shift towards understanding health from a holistic perspective as well as increased recognition of public health’s contributions to the health of the population [3]. Outside of global public health emergencies, such as Ebola or Zika Virus, attention to the role that public health plays in the protection and advancement of health has often taken a backseat to discussions of healthcare reform [4, 5]. For many, health and the health system equate to healthcare, namely clinics and hospitals. In the context of the United States and Canada, considering, for example, that medical care consumes the largest amounts of the health budget in Canada and the United States, it is unsurprising that there is generally little public or political interest in strengthening or investing in public health systems [6, 7]. For example, amid concerns that public health across Canada continues to be weakened through budgetary cuts and lack of investment in public health infrastructure, there remains little evidence related to understanding public health systems or what is currently done in practice in a comparative fashion [6, 8]. Most public health research has focused on the evaluation of programmes aimed at individual or population-level interventions and understanding the causes and patterns of risk of ill health and disease rather than informing broader questions about the organisation, delivery or funding mechanisms of public health systems [7, 9–11]. Amid the current novel coronavirus disease (COVID-19) pandemic, understanding how public health and broader health systems function, is crucial.

Health services and system researchers have not adequately acknowledged public health as a vital component and contributor to health systems, and achievements made by public health activities, such as communicable and non-communicable disease control, are often attributed to the delivery of primary healthcare services and advances in biomedical interventions [12]. While multiple health systems frameworks identify the components, functions and goals of healthcare systems, no clear or consistent definition of public health systems exist [13, 14].

Defining public health systems can help determine how to best design systems and deliver programmes and services to support public health within the larger health system and other key institutions and move discussions about the relationship between public health and healthcare forward. As a first step, this paper addresses a priority research area that called for the development of a framework describing the key elements of public health systems [3, 15, 16]. A qualitative synthesis of the current literature was completed to investigate how public health systems have been defined and classified as well as the differences between healthcare systems and public health systems within established conceptual frameworks for health systems.

## Methods

This qualitative review adopted the critical interpretive synthesis (CIS) approach as the overarching methodology while using a second and complimentary qualitative strategy, the best-fit framework synthesis (BFF), to guide structured data extraction and analysis. CIS differs from traditional systematic reviews in several ways; namely, (1) it is an iterative process that explicitly allows for the critical re-interpretation of existing literature and filling of conceptual gaps, and (2) it prioritises the inclusion of papers based on relevance to the research question, including grey literature, increasing the likelihood of capturing relevant documents [17–19]. The BFF is used to test, refine and/or generate relevant frameworks, theories or conceptual models using systematically retrieved empirical data. For this study, BFF was determined to be useful for the organisation, extraction and analysis of large amounts of data, as a priori or pre-identified codes allows researchers to utilise and generate codes and interpret themes but not be restricted by an existing framework, model or theory [20]. This study conformed with the recommended PRISMA guidelines [21] and was registered in PROSPERO (CRD42016049967).

### Search strategy

Following pilot testing, the databases searched included EBSCOhost, OVID, Scholars Portal, Web of Science, Cochrane Library and Health Systems Evidence. The final search was conducted on 25 October 2016. Studies were not limited to date, language or study design. Additional sources were identified through reviewing the references of included documents to find relevant material and through internet searching to fill conceptual gaps using non-systematic searching. The initial search strategy can be found in Additional file 1. As public health systems have not been clearly defined, our search strategy sought to include terms that may be used interchangeably within the literature but warrant clear definitions. For the purpose of this study, we define a system as “*a set of inter-connected parts that have to function together to be effective*” [22], a framework as “*a basic conceptual structure*” [23], a model as “*a standard or example for imitation for comparison*” [24], and classification as “*an arrangement of people or things into groups based on ways that they are alike*” [25].

### Study selection

Records identified were screened, duplicates were removed, and titles and abstracts were independently reviewed for exclusion by two reviewers. Records were excluded that (1) did not describe local, state/provincial/territorial, or national public health systems, frameworks or critical components, (2) addressed publicly funded healthcare systems, unless it also addressed the role of public health, and (3) were in languages other than English, French or Spanish. Records were not restricted by date or country as we wanted to obtain a general picture of public health systems globally. To help address the compass question and maximise the diversity of papers, potentially relevant documents were purposively sampled and prioritised for inclusion if they were clearly relevant to the research topic, offered conceptual insights about full frameworks, and were able to provide a cross section of different jurisdictions [19, 26]. Full-text documents were retrieved and assessed for eligibility with additional documents found through reference chaining of all included studies or internet searches to help fill conceptual gaps.

### Data abstraction

A data extraction tool was developed to organise the key themes of relevant documents and bibliographic information, including title of document, author(s), study type, context of study, key topic areas, and further relevant references from paper. Documents were imported into NVivo 11 software to facilitate the coding and organisation of data. Seven documents were randomly selected and coded independently by two reviewers to ensure consistency. Disagreements were resolved by consensus.

### Data analysis and synthesis

A current leading health systems and policy classification scheme, the health systems arrangements framework (based on the three key building blocks of governance, financial and delivery arrangements), was used to form the initial a priori codes [27]. Originally developed as a taxonomy of health system topics to classify documents for Health Systems Evidence, this framework was chosen as the BFF theoretical framework because it is comprehensive (e.g. includes the essential components of WHO’s health system building blocks [22]), easy to understand, and has been used in various international contexts for health systems and policy research and applied work, for example, to develop health systems guidance documents and to contextualise research for evidence-based decision-making in Peru and Uganda [28–30]. Codes were added inductively from the data and were linked into themes. Data analysis continued until there was data saturation and conceptual gaps were addressed [31].

## Results

### Search results and study selection

**Fig. 1:**
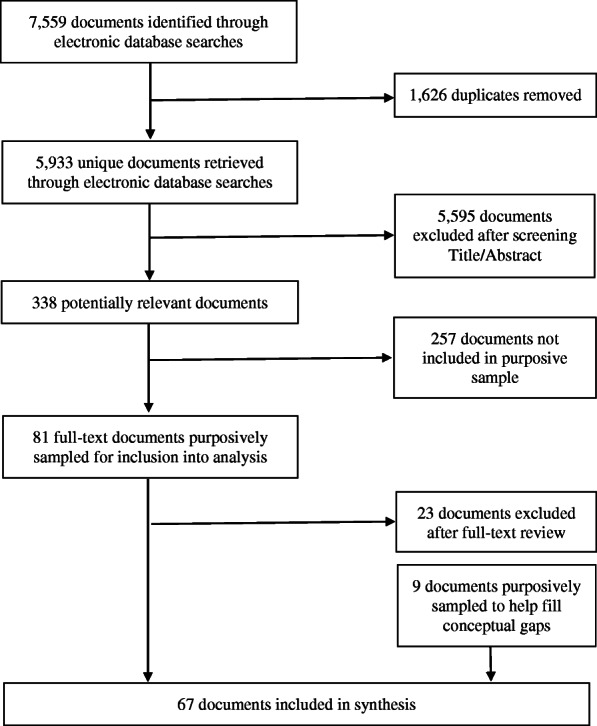
PRISMA flow chart for inclusion/exclusion of documents [21]

Electronic database searches identified 5933 unique citations, 338 of which met inclusion criteria after title and abstract exclusion; 81 of these documents were purposively sampled and full-text review excluded 23 records. Nine additional documents were attained through reference chaining and internet searches. In total, 67 documents were included (Fig. 1). A description of these documents can be found in Additional file 2.

The results are presented in four sections – defining public health and public health systems; roles and functions of public health; public health systems; and public health within health systems. High-level findings are presented in the text and more details are provided in the Additional files.

### Defining public health and public health systems

Most documents defined public health via its functions; therefore, separate sections were created for defining public health and public health systems and for describing the roles and functions of public health.

#### Public health

Seven definitions of public health were found (Additional file 3). Public health was described as a multidisciplinary area of practice, concept and set of values that engaged in a larger population health perspective. Eight documents used the definition of public health provided by WHO as an art and science whose organised efforts aim to prevent illness and disease as well as to protect and promote health within society [32–39]. Five other definitions expanded or emphasised various priorities within public health practice and included values of equity and equality.

#### Public health systems

Public health systems were defined in 20 documents, with 10 unique definitions identified (Additional file 3). Eleven documents defined public health systems as all levels of governmental and non-governmental entities which share in the responsibility for ensuring healthy social and physical environments, and consist of a variety of organisations that contribute to the core functions of public health to protect and promote health within the community [12, 40–49]. Public health systems were also defined based on their composition, level of service, contributing actors, mission and activities, or a combination of these. Public health was largely seen as a governmental responsibility and included partnerships between formal (government) and informal (private sectors, volunteer) organisations.

### Roles and functions of public health

#### Frameworks

Subsystem models of delivery, governance, finance, and roles and functions were identified, for example, Mays et al.’s [45] typology of public health delivery systems, but none provided a comprehensive public health system framework. Several frameworks identified essential public health functions (Additional file 4). The most frequent frameworks were the Institute of Medicine’s three ‘core’ public health functions of assessment, policy development and assurance [12, 38, 42, 47, 48, 50] and the ‘Ten Essential Public Health Services’, which were developed to further refine the specific set of functions and services within public health systems in the United States but have been adapted elsewhere [40, 42, 44, 48, 51–56].

#### Roles and functions

Thirty-nine documents defined or highlighted what they identified as the ‘essential’ functions of public health. Additional file 4 provides a table as a way of organising what functions were found within the literature across a variety of countries, following the three core public health functions and 10 essential services. The following were listed as public health functions and services in more than half of the documents: health promotion (*n* = 30); health protection, which included air, water, and food quality and inspection as well as environmental and occupational health activities (*n* = 26); investigation and surveillance (*n* = 25); emergency planning, preparedness and response (*n* = 25); health assessment and monitoring (*n* = 24); injury and chronic disease prevention and management (*n* = 23); and linking with and providing personal clinical services, which included maternal and child health services, minority, rural, indigent, mental, clinical and community health improvement activities, to targeted and/or vulnerable populations (*n* = 22). Communicable disease control (*n* = 18); research (*n* = 16); regulation and enforcement (*n* = 16); resource and organisational management, including leadership, governance capacity, resource management and development of organisational structures (*n* = 14); establishment of partnerships and advocacy in communities (*n* = 13); evaluation of health services (*n* = 11); policy development and planning (*n* = 11); workforce strengthening (*n* = 9); programme implementation (*n* = 4); laboratory services (*n* = 3); hospital and long-term care facility licensing (*n* = 2); and vital statistics (*n* = 2) were also identified as being the responsibility of public health in various jurisdictions. Functions and services had to be interpreted and summarised, as there were different terms being used to represent the same activities between jurisdictions. For example, health protection and environmental health were both used to describe the responsibility for testing and monitoring the quality of air, food and water, and population health assessment was used to describe monitoring, surveillance or epidemiological activities.

### Public health systems

It was found that public health system descriptions fit the health system arrangements framework well, with the addition of partnerships and communication, which affected each of the other parts of the system (Additional file 5). The health systems arrangements framework was refined to highlight differences between terms and components of public health systems (Table 1). While little to no evidence on certain features, such as commercial authority, remunerating providers and incentivising consumers, was available within the literature, these arrangements are still applicable to public health, and thus remain within the public health systems framework.

**Table 1:** Public health system arrangements, adapted from health system arrangements framework

Key features		
Partnerships and Communication	Governance arrangements	Policy authority
		Organisational authority
		Commercial authority
		Professional authority
		Consumer and stakeholder involvement
	Financial arrangements	Financing systems
		Funding organisations
		Remunerating providers
		Purchasing products and services
		Incentivising consumers
	Delivery arrangements	How are programmes and services designed to meet consumers’ needs
		By whom are programmes and services provided
		Where are programmes and services provided
		With what supports are programmes and services provided

#### Governance arrangements

##### Policy authority

Four levels of policy authority were identified within the public sector, namley international, national, state/provincial/territorial, and local. The degree of decentralisation within a country or state/province determined the responsibilities and structural organisation of agencies within public health systems [4, 12, 32, 34, 37, 44, 46, 49, 57–60]. Most national public health agencies were primarily responsible for providing guidance and acting as a source of expertise while giving states/provinces authority to organise public health [4, 32, 39, 48, 54, 57, 61–64]. Many state/provincial governments established overall priorities, strategic direction, policies, strategies, standards, and funding models for local public health agencies [4, 34].

##### Organisational authority

Regional or local health units planned and implemented the majority of services, developed policies and communicated legislation [34, 57, 61, 63, 65].

##### Consumer and stakeholder involvement

In public health systems, consumers most often referred to targeted populations and communities rather than individuals, as is more common in healthcare systems. Stakeholders included other public sectors, communities, service providers in and outside of the health system, the private sector, and individuals [63]. Community partnerships and public engagement were identified as being important for individual and community health, accountability, and an influential factor in the operation of local public health agencies [33, 41, 47, 56, 61, 63, 66, 67].

#### Delivery arrangements

In public health systems, the terms ‘programmes’ or ‘services’ seemed to better reflect the wide range of activities and roles of public health within the larger health system than the term ‘care’.

##### How are programmes and services designed to meet consumers’ needs

Public health functions were carried out by all levels of government, including federal, state/provincial/territorial and local, but most activities remained organised at the state/provincial level or locally in many countries [2, 38–40, 48, 58, 59, 62, 63, 68–71]. Delivery of public health services often rested at the local level but, in some instances, were delivered at the state/provincial level or through separate government or private organisations [38, 48, 59, 62]. In one United States-based example, although public health and healthcare were largely independent of one another, public health increasingly provided personal health services for pre-identified or vulnerable groups [72].

##### By whom are programmes and services provided

Most public health programmes/services were provided by public sector employees as part of a public health unit, as well as faith-based groups, private businesses, social services agencies, schools, workplaces and healthcare providers [46, 73]. Healthcare and other sectors support public health in its missions by participating in surveillance, health protection and emergency planning activities [37, 73, 74]. Because of the diversity in the organisations and people involved in providing public health programmes and services, the size of a public health workforce is difficult to determine [5, 57, 63, 73, 75].

##### Where are programmes and services provided

Delivery of public health programmes and services occurs in multiple public and private settings, including schools, homes of private citizens, workplaces, clinics, public health laboratories, local public health agencies and offices, and various indoor and outdoor spaces within the community [57, 61]. Partnerships and contracts with non-governmental and community organisations in public and private sectors have often been established to circumvent barriers to service provision (e.g. due to geographical location or size of jurisdictions) [40, 49, 71].

##### With what supports are programmes and services provided

Support was often referred to as capacity in human health resources [2, 40, 51, 57, 59] and information technology [62, 75]. A few articles discussed the use of technology as a tool used to deliver and support public health activities and messaging, and included services such as eHealth, web portals, mobile phone applications and social media [66].

#### Financial arrangements

It was difficult to estimate the direct and indirect financial contributions by public and private sectors given the diversity in public health activities [39].

##### Financing systems

Several sources estimated that, on average, public health systems received between 3% and 8% of the national health budget [5, 32, 38, 39, 44, 51, 57, 72]. Like healthcare, public and private funding sources existed in these systems, with many being publicly financed through general taxation, including federal, state/provincial and local taxes such as income, property and sales taxes [32, 39, 47, 52, 57–59, 62, 69, 76]. Private sector financing included out-of-pocket service fees and for-profit and non-profit organisations [44, 47, 52, 59]. A significant part of public health funding is derived from external donors, particularly for disease-specific initiatives, in low-income countries [58].

##### Funding organisations

Revenue transfers from national governments to state/provincial or local public health agencies, with funding being distributed to local health agencies to deliver services, were most prevalent. Funding was largely allocated by funding formulas; however, a combination of funding mechanisms, such as activity- and standard-specific funding and reimbursements, per capita allocations, competitive and needs-based grants, and performance-based funding were also reported [38, 46, 47, 55, 58, 59]. Other sources of funding originated from other public sector partners and from collaborations between public and private sectors [39, 47, 59, 65]. In some instances, external donors allocated funds to community-based organisations to target specific community health needs or provided informal funding for ‘non-essential’ public health programmes [4, 12, 39, 47, 63, 65].

##### Purchasing products and services

Funding organisations and purchasing products and services were strongly linked. Many federal and state/provincial governments allocated funds for specific public health activities, which influenced the availability of services [38, 46, 47, 58, 59]. Generally, there was a trend towards a substantial portion of public health funding directed at individual clinical services (e.g. maternal and child health, mental health, prenatal visits, family planning) [12, 60].

#### Partnerships and communication

Partnerships were identified as an essential way to extend the reach of programmes to target population health issues and to share expertise, information and resources [2, 4, 39, 40, 46, 56, 59, 65, 66, 68, 77–81]. Partners included other local, national and international government agencies, the healthcare system, academic centres, private sector businesses, faith groups, foundations, service organisations and communities [32–34, 40, 45, 46, 48, 52, 54, 56, 62–64, 72, 79, 82, 83]. Within governance arrangements, the goal of partnerships was identified as community empowerment and capacity-building for successful interventions [4, 56, 61, 74, 79, 82, 84]. Engagement within communities reportedly increased stakeholder involvement in policy and decision-making [82, 84].

Public health is an information-dependent sector that requires constant information exchange in order to support public health functions, activities and policies, especially in emergency planning and response [2, 58]. Communication not only improves surveillance and response systems between all levels of government and internationally but is necessary for effective knowledge translation activities [5, 84, 85]. Clear, consistent and timely communication is essential for delivering messages to the public, preventing mixed messages and encouraging public engagement. Current and evolving technology, such as the internet and other mass media sources, are tools that support this effort by improving health literacy and outreach [66] but can also spread misinformation.

### Public health within health systems

In framing how public health is seen as part of a larger health system, the literature pointed to two related but separate concepts – that of system integration and the role of public health in promoting a population health approach.

Sofaer [79] states that the best way to judge how effective a health system is, is by how well it can improve the health of individuals and populations. Interest in integrating public health and healthcare systems is not new [34, 48, 76]. While definitions of integration vary, integration in this report is the relationship between public health and healthcare and the extent to which services are provided to promote and achieve health. Integration is believed to bring the two systems closer together to provide a seamless service delivery within the larger health system and better respond to the needs of both individuals and communities [33, 65]. Potential benefits of integration include bringing a population health perspective to the healthcare system, increased access to care, and the reduction of direct and indirect healthcare costs [4]. However, the literature also points to various challenges regarding integration and what it might mean for the future of the public health system. These include the loss of public health authority and expertise, capacity and management of competing priorities, consequently linked to adverse health outcomes [58]. Over time, the diversion of public health resources to primary care, loss of positions in public health units, and loss of linkages to community partners and communities would hinder public health from being able to extend the reach of its activities and lead to fragmentation in programme delivery and in the services necessary to protect the health of the population such as community health assessments, programme planning, and disease control and surveillance [9, 58, 86].

**Fig. 2:**
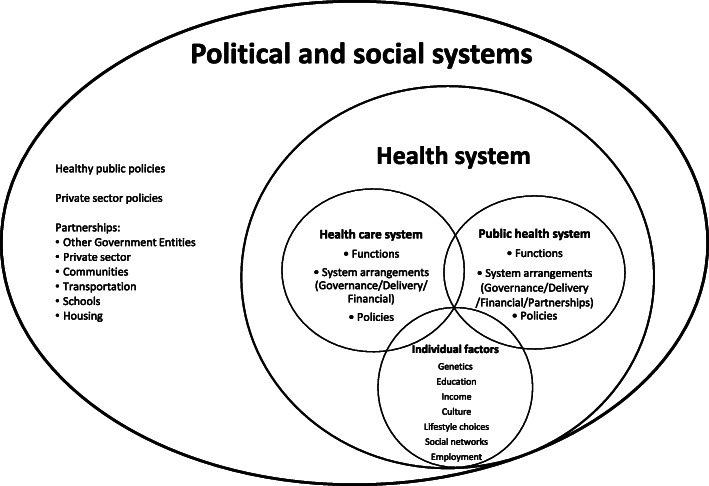
Conceptual fit of public health systems within current health systems

From the literature, healthcare and public health are separate systems, often with their own aims and functions, governance and financial systems, and ways of delivering services, although significant overlap has been observed, particularly within delivery arrangements. The health system is separate from but influenced by the larger political and social systems. Health, within this health system, is determined by individual factors and access to and use of public health and healthcare services. Yet, the wider determinants of health recognise the importance of social and political factors on health. Figure 2 aims to organise how public health currently fits within a health system. It is important to note that public health is often equated to and touted as a steward of population health. Population health is the driving force behind public health. Its upstream focus, following an ecological model of health, is concerned with how individual, social and environmental determinants influence health outcomes [53, 55]. A strength of the population health approach is that it recognises that people are not passive but are active participants in their own health outcomes. Individual health is supported by both public health and healthcare activities and by how individuals interact with these systems and their larger social environments. There is a constant exchange between individuals, healthcare, public health, and the political and social systems they are embedded in, with more resources, programmes and services targeted towards those identified as vulnerable to try and establish a level of equity in health outcomes. It could be argued that, while activities in public health are population based, the ultimate target of public health is still to support individual health within the larger community. For example, although health promotion messages and activities are delivered to the population, the goal of these activities is to encourage individuals within communities towards healthier lifestyles (e.g. tobacco cessation, vaccinations), whose health statistics are then tracked (e.g. surveillance) and regarded as the population’s health.

Population health is conceptualised as extending far beyond the health system to include the political and societal contexts. While policies outside of the public health system may not be implemented to directly impact population health, they often do. For example, taxes on carbon emissions have short- and long-term effects on population-level health outcomes. Similarly, public health systems affect, and are affected by, many sectors. As broader determinants of health are becoming increasingly recognised as influential, there has been an increased emphasis on holistic approaches to healthy public policies [9]. Recent work has focused on holistic approaches to health such as Health in All Policies and One Health. Health in All Policies refers to the intersectoral aim of integrating health considerations into the actions, interventions and policies outside of the health sector, and One Health refers to the approach that recognises that human health is influenced by both animal and environmental health [87, 88]. Figure 2 highlights the gaps that exist between what we currently have, at least in high-income countries, and paradigms of population health, Health in All Policies and, especially, One Health. For example, public health is often separate from healthcare and from the political process (i.e. healthy public policies). Population health spans further than public health’s reach and integration may need to be reconceptualised to align with a broader vision of health.

## Discussion

### Main findings

The synthesis suggests that public health systems have not been clearly defined because (1) public health systems have been conceptualised in various ways and (2) there is overlap in terminology with publicly funded healthcare systems. One further potential reason for the lack of clarity regarding definitions and the change in the use of terms over time could be related to funder and publication preferences. No comprehensive public health system frameworks were identified within the literature although there was significant emphasis on defining the essential roles and functions of public health. These are broad and consensus on essential functions is often absent between jurisdictions, which made comparisons challenging. Services not provided by healthcare systems are often taken up by public health, increasing pressures on already limited budgets. In addition, response to health emergencies appears to have largely been adopted by public health systems because they are most likely to possess the capacity and expertise to organise and respond to large-scale events or threats to health. We found that, while many components of public health systems fit under the governance, delivery and financial arrangements of traditional healthcare systems, there are noted differences, specifically related to the role of partnerships and communication within public health. Partnerships provided the structure for multi-sectoral collaboration and facilitated communication and information exchange to accomplish the core functions of public health. A proposed framework for public health systems is presented in this paper.

The argument is made that public health and healthcare share the common goal of supporting the health of individuals within populations. Integration, the intersection of public health and healthcare, is believed to bring the two systems closer together to provide seamless service delivery within the broader health system that better responds to the needs of both individuals and communities [33, 65]. The challenge with developing these integrated health systems is determining how to best align financial, governance and delivery arrangements, ensuring both complementarity and positive health outcomes. As population health extends beyond the health system to include the political, environmental and societal contexts, as such, it is important to understand these larger contexts within which health systems operate [89]. The conceptualisation of the current fit of public health systems within health systems has two important ramifications. The first being that integration of healthcare and public health will be difficult at best given that the aims, governance, finance and service delivery are not often aligned. There would have to be significant incentives for integration to happen and even with that, there may not be a shared vision of health to drive collaboration between these systems. The second ramification is that public health will not be able to inform healthy public policies unless they have a seat at the decision-making table for policies outside of the public health realm. There may be arguments on both sides as to whether this is practical or desired, but the case can be made that population health, encompassing the broader social determinants of health, will not occur within the current paradigm and structures.

### Strengths and limitations

The flexibility of the CIS approach allows for a broad sampling frame and iterative filling of conceptual gaps. The BFF approach provides a structured approach to data analysis but also for change if a more applicable model is identified. The combination of these two qualitative approaches allowed for a broad research question in an area that is not well defined and helped bring a lot of data together in an efficient manner. The study was informed by a diverse team of experts in public health, health systems research and qualitative research methodology. The search strategy may not have captured all terms and concepts regarding public health systems. To try and mitigate this, a search string was developed with broad search terms to identify as much relevant literature as possible. Additionally, as the reviewed literature mainly covers the period from 2000 to 2016, the search strategy may not have captured all relevant documents such as recent institutional reports. While literature addressing health systems have origins before 2000, more recent documents were purposively sampled and prioritised in the document selection process as they would expectedly include earlier relevant works. This also presents an opportunity to review works after 2016, such as the 2018 report on the organisation and financing of public health services in Europe, and more recent documents that highlight the importance of improving population health through the Sustainable Development Goals (SDGs) [90, 91]. CIS requires constant reflexive analysis by the principal investigator and results may vary if another person were to replicate this study; however, the use of a priori codes was used to increase transparency. Finally, although public health systems from various countries were reviewed, almost all documents were from high-income countries, mostly originating from the United States and Canada. While some of the results may be equally relevant to systems outside of these Western contexts, the findings highlight the need for future research outside of these jurisdictions, for example, in low- and middle-income countries, particularly in light of the SDGs.

### Placing this work within the literature

This study is a first attempt at defining a holistic public health systems framework and highlighting the differences and similarities between public health and healthcare system arrangements. We have specifically addressed a priority research theme proposed by Canadian and United States federal agencies, such as the Canadian Institutes of Health Research Institute of Population and Public Health, the Centers for Disease Control and Prevention, and other stakeholders, to describe dimensions of public health systems and conceptualise a framework for public health systems.

### Practice and policy implications

This study suggests five considerations for practice and policy. First, defining public health systems solidifies and challenges public health’s role to encourage political interest to secure the investment necessary to improve health system capacity. The COVID-19 pandemic has demonstrated the importance of understanding the role of public health within the larger health system, particularly with respect to the capacity to respond to public health crises efficiently and effectively. As was observed following the 2004 SARS pandemic, it is expected that there will be an increase in renewed calls and discussion around public health systems strengthening. Second, the discourse around establishing essential functions of public health is enhanced. Our synthesis has identified a growing concern that public health is currently filling gaps within the healthcare system by providing clinical services to targeted or vulnerable groups, consuming vast amounts of both human and financial resources from already under-resourced public health systems [5, 60, 69]. Thus, defining public health and the boundaries of public health systems could be an important step towards measuring performance and preventing public health systems from becoming too overburdened from the increasing scope of public health clinical activities [48, 54]. Third, this study has reinforced the importance of partnerships in the work of public health. Partnerships have the potential to form and navigate systems in contexts challenged by limited resources. For example, the United Nations Millennium Development Goals, aimed to tackle societal issues influencing health, such as poverty, education, and gender equality and, while substantial progress was made, the state of many health systems revealed barriers to reaching specific targets and delivering services to the most vulnerable, particularly for those in many low- and middle-income countries. The SDGs, whose agenda is broader and more ambitious, explicitly recognise the broader determinants of health, by establishing social, economic and environmental objectives such as climate action, sustainable cities and communities, economic development, and social inclusion [92]. Defining public health systems serves as a building block for under-developed or transitioning public health systems and services, whereby determining roles and functions of public health systems allows practitioners to identify areas that require strengthening. Fourth, this synthesis suggests that the critical differences between public health and healthcare systems need to be acknowledged and negotiated for integration to be successful. The gaps that exist between the public health and healthcare subsystems have been highlighted. Lastly, the idea that public health is the champion of population health is presented as a challenge. Population health is influenced by political and social factors outside of the public health system. The idea of public health as the steward of population health requires serious consideration, especially if public health continues to be excluded from the decision-making process and its role in protecting and promoting health is relegated to the background.

### Research implications

By developing a method that combined the best aspects of two qualitative systematic review methods, CIS and BFF, we were able to bring substantial amounts of data together in a timely manner, while simultaneously testing and refining a well-known framework in a critical way. The use of other frameworks, such as the performance-based conceptual framework by Handler et al. [42], or an assessment framework identified by Martin-Moreno et al. [53], provide other ways to examine public health systems and could be a way to validate the findings of this study or illustrate different health system ideas. The performance of public health systems cannot be measured if definitions, functions and key components are not well defined. The numerous variations in terminology make it difficult to perform a comparative analysis of public health systems across jurisdictions. Similarly, the differences in defined functions, or a lack thereof, limits our ability to monitor quality indicators between systems. Furthermore, the lack of research in public health and public health systems hampers both interest and investment in public health and limits the development of recommendations for evidence-based practice. Further research is required to determine what integration might look like and at what systems levels integration might work best. This presents additional opportunities for future research, particularly with respect to the gaps highlighted within financial arrangements such as remunerating providers and incentivising consumers. Lastly, this study is a first attempt at trying to understand how public health systems have been conceptualised. A public health systems framework (Table 1) and conceptualisation of how public health currently fits within the larger health system (Fig. 2) are proposed and can be applied and tested in real life settings as well as used to guide further research and practice in public health systems.

## Conclusion

The aim of this paper was to examine the literature on how public health systems have been defined and classified and to illustrate how current public health systems align within established conceptual frameworks for health systems. Defining the scope of public health systems is crucial to solidify public health’s role as part of the larger health system and the degree to which public health and healthcare systems are different should be understood if public health is to be able to attend to its primary mandate within integrated health systems. While there is increased movement towards health systems focused on population health, specifically the increased focus on Health in All Policies and One Health, many gaps exist to reach those aims.

## Supplementary information

**Additional file 1.** The initial search strategy and databases. Additional file 1 provides search strings and detailed database search strategy.

**Additional file 2.** Characteristics of documents reviewed for this study. Additional file 2 provides a description of the characteristics of the documents included in this study.

**Additional file 3.** Definitions of entities and systems. Additional file 3 provides the definitions found within the literature regarding public health and public health systems.

**Additional file 4.** Public Health Functions and Purpose. Additional file 4 provides a table as a way of organising what functions were found within the literature across a variety of countries, following the three core public health functions and 10 essential services.

**Additional file 5.** Aligning public health systems into the health system arrangements framework. This additional file includes a summary of the coded data sources used to align public health within the health system arrangements framework.

## Data Availability

All data generated or analysed during this study are included in this published article and its additional files.

## Abbreviations

BFF: Best-fit framework synthesis
CIS: Critical interpretive synthesis
SDGs: Sustainable Development Goals
