# Supplementary material for: Defining and classifying public health systems: a critical interpretive synthesis
Source: Health Res Policy Syst. 2020 Jun 16;18:68. doi: 10.1186/s12961-020-00583-z (PMC7296190; doi:10.1186/s12961-020-00583-z)
Supplement: Supplementary file 1 — Additional file 1. The initial search strategy and databases. Additional file 1 provides search strings and detailed database search strategy. [file 12961_2020_583_MOESM1_ESM.pdf]

Appendix 1: Database Search Strategy

Database	Database name	Description of database	Search string used	Number found and comments
EBSCOHost	AgeLine	Covers issues of aging over 50+ from health sciences, policy and economics perspectives, among other	**Title**: Public health; **All Text**: system*; **All Text**: (deliver* OR governance OR organization OR classif* OR structure* OR manag* OR fund* OR function* financ* OR role OR purpose OR typology OR framework* OR model* OR component* OR definition*)	2, 003
	CINAHL	Includes allied health		
	Social Sciences Abstracts	Applied and theoretical aspects of social sciences		
Scholars Portal			**Article Title**: Public health; **Article Title**: system*; **Anywhere**: (deliver* OR governance OR organization OR classif* OR structure* OR manag* OR fund* OR function* financ* OR role OR purpose OR typology OR framework* OR model* OR component* OR definition*)	414
OVID	Global health	Includes public health topics in an international forum	**Title**: Public health; **Heading words**: system*; **All fields**: (deliver* OR governance OR organization OR classif* OR structure* OR manag* OR fund* OR function* financ* OR role OR purpose OR typology OR framework* OR model* OR component* OR definition*)	960
	Ovid Healthstar	Clinical and non-clinical aspects of healthcare delivery		

Web of Science	Core collection	Sciences, social sciences, arts, humanities and includes gray literature	**Title**: Public health; **Topic**: system*; **Topic**: (deliver* OR governance OR organization OR classif* OR structure* OR manag* OR fund* OR function* financ* OR role OR purpose OR typology OR framework* OR model* OR component* OR definition*)	3, 356
Cochrane Library	Cochrane Library	Includes systematic reviews, methodology reviews, clinical trials and others relating mostly to healthcare	**Title, abstract, keywords:** Public health; **Keywords:** system*; **Search All Text:** (deliver* OR governance OR organization OR classif* OR structure* OR manag* OR fund* OR function* financ* OR role OR purpose OR typology OR framework* OR model* OR component* OR definition*)	305 – 297 downloaded correctly
Health Systems Evidence	Health Systems Evidence	Health systems database	Public health; system*; (deliver* OR governance OR organization OR classif* OR structure* OR manag* OR fund* OR function* financ* OR role OR purpose OR typology OR framework* OR model* OR component* OR definition*); Filtered by: Sectors: Public Health; Any system arrangement; Document Features: Health reform descriptions; Health system descriptions; Intergovernmental organizations' health systems documents; Canada's health systems documents; Ontario's health system documents; Target: Health System	529 – saved in Excel, could not save into Zotero or Refworks. Manually reviewed for duplicates and inclusion/exclusion separately.
TOTAL				7, 559
