# Supplementary material for: Defining and classifying public health systems: a critical interpretive synthesis
Source: Health Res Policy Syst. 2020 Jun 16;18:68. doi: 10.1186/s12961-020-00583-z (PMC7296190; doi:10.1186/s12961-020-00583-z)
Supplement: Supplementary file 2 — Additional file 2. Characteristics of documents reviewed for this study. Additional file 2 provides a description of the characteristics of the documents included in this study. [file 12961_2020_583_MOESM2_ESM.pdf]

Appendix 2: Characteristics of documents reviewed for this study

Characteristics		Number n=67	Percent (%)
Peer-reviewed vs. gray literature	Peer-reviewed	51	76
	Gray literature	16	24
Peer-reviewed	Conceptual	26	51
	Empirical	25	49
Design (for conceptual papers)	Discussion paper	11	42
	Non-systematic review	7	27
	Commentary	5	19
	Theory paper	2	8
	Editorial	1	4
Design (for empirical papers)	Cross-sectional	9	36
	Qualitative	9	36
	Case study	3	12
	Systematic review	2	8
	Cohort	1	4
	Mixed methods	1	4
Context	Global	7	10
	Regional	3	5
	National	43	64
	State/Provincial	12	18
	Local	2	3
Year of publication	2015-present	9	13
	2010-2014	13	19
	2005-2009	22	33
	2000-2004	17	25
	before 2000	6	9
