# Supplementary material for: Defining and classifying public health systems: a critical interpretive synthesis
Source: Health Res Policy Syst. 2020 Jun 16;18:68. doi: 10.1186/s12961-020-00583-z (PMC7296190; doi:10.1186/s12961-020-00583-z)
Supplement: Supplementary file 3 — Additional file 3. Definitions of entities and systems. Additional file 3 provides the definitions found within the literature regarding public health and public health systems. [file 12961_2020_583_MOESM3_ESM.pdf]

Appendix 3: Definitions of entities and systems

Entity	Definitions/Descriptions	Citation	Suggested definition
**Public Health**	The art and science of health promotion and protection, disease prevention, and the improvement and prolonging of quality of life through the organized efforts of society.	(32–39)	Public health is an art and science, based on objective findings but responsive to the needs and contexts of populations, concerned with addressing the health needs of a community. It is a diverse set of organized activities aimed at improving quality of life and reducing health disparities to enable people to thrive.
	The organized efforts of society to prevent morbidity and premature mortality, keep people healthy, improve health and well-being, and reduce health inequalities.	(66)	
	The proactive approach to protecting the health of a community.	(93)	
	Public health fulfills society's collective interest in assuring environments that allow people to thrive.	(72)	
	The political art of applying science with the aim of reducing health inequalities while ensuring the overall health of the population.	(58)	
	Social medicine is the impact of decisions or policies made by other sectors that impact health, i.e. welfare, education	(79)	
	The diverse set of activities that focus on the promotion and protection of the health of the population and address health needs.	(77)	
**Public Health System**	Includes all levels of governmental and non-governmental entities that share in the responsibility for ensuring healthy environments. It is a complex network of organizations that contribute to the core functions of public health to protect and promote health within the community.	(12,40–49)	A public health system is the collective capacity of governmental, private, and other public sector entities that support the mission and core functions of public health. It is the cumulative arrangement of resources, infrastructure, and policies impacting health that exist to support public health within communities.
	The public health system consists of national, state/provincial, and local agencies.	(38)	
	Governmental public health agencies that partner and interact with other public and private entities to engage in a variety of public health activities within communities.	(54)	

	Governmental, private, and public sector agencies and organizations whose actions impact the health of the population, as well as infrastructure and laws that support public health activities.	(52)	
	Governmental, non-governmental and community organizations that operate at all levels of government and are responsible for program delivery, policy setting, funding and the coordination of public health initiatives.	(34)	
	Public health systems provide services to the population with the primary goal of reducing exposure to disease through regulations and education.	(64)	
	Individuals and organizations that work towards the health of a community or population, usually revolving around a government agency that directs the actions of partners to accomplish system goals.	(94)	
	Public health systems work at the local, regional, national and international levels to deliver comprehensive programs through partnerships and multidisciplinary teams of practitioners, specialists, and advocates to improve and protect health in communities.	(83)	
	The public health system is separate and complimentary to the healthcare system. Due to the nature of the public health, public health systems consist of essential partnerships between formal and informal public health organizations and societal groups to influence determinants of health.	(2)	
	The essential building block of public health that brings together community and organizations through partnerships to perform essential public health functions, standardizing public health practice and performance.	(67)	

Additional References

93. Benton K, Polite S. The Disconnect between Public Health and Health Care. Health Prog. 2016;97(2):58–61.

94. Halverson PK. Embracing the strength of the public health system: why strong government public health agencies are vitally necessary but insufficient. J Public Health Manag Pract. 2002;8(1):98–100.

95. Salinsky E, Gursky EA. The case for transforming governmental public health. Health Aff (Millwood). 2006;25(4):1017–28.

96. Chambers LW, Sullivan SM. Reflections on Canada's public health enterprise in the 21st century. Healthc Pap. 2007;7(3):22–30.

97. Deber R, McDougall C, Wilson K. Public health through a different lens. Healthc Pap. 2007;7(3):66–71.

98. The Chief Public Health Officer's report on the state of public health in Canada, 2008. [Internet]. The Chief Public Health Officer's report on the state of public health in Canada, 2008. 2008. Available from: http://www.phac-aspc.gc.ca/cpho-acsp/index-eng.php

99. Mays GP, McHugh MC, Shim K. Institutional and Economic Determinants of Public Health System Performance. Am J Public Health. 2006;96(3):523–31.
