# Supplementary material for: Defining and classifying public health systems: a critical interpretive synthesis
Source: Health Res Policy Syst. 2020 Jun 16;18:68. doi: 10.1186/s12961-020-00583-z (PMC7296190; doi:10.1186/s12961-020-00583-z)
Supplement: Supplementary file 4 — Additional file 4. Public Health Functions and Purpose. Additional file 4 provides a table as a way of organising what functions were found within the literature across a variety of countries, following the three core public health functions and 10 essential services. [file 12961_2020_583_MOESM4_ESM.pdf]

Appendix 4: Public Health Functions and Purpose

Region			Source	Framework																		
	Country	State/ Local		I.O.M. Three Core Functions of Public Health (U.S.A.)										Established Essential Services								
			(12,38,42, 47,48,50)	Assessment		Policy Development			Assurance													
			(40,42,44, 48,51–56)	10 Essential Public Health Services (U.S.A.)																		
				Health Assessment and Monitoring	Investigation/Surveillance	Inform/educate/Health Promotion	Partner Engagement & Advocacy	Policy Development & Planning	Regulation/ Enforcement	Link & provide health services	Workforce strengthening	Evaluation of health services	Research	Communicable Disease Control	Chronic Disease & Injury Prevention	Health Protection	Emergency Planning & Response	Laboratory Services	Licensing	Program Implementation	Resource & Organization Mgmt.	Vital Statistics
North America																						
	U.S.A.		(40,42,44, 48,51–56)	**x**	**x**	**x**	**x**	**x**	**x**	**x**	**x**	**x**	**x**									
			(45)		**x**	**x**									**x**	**x**	**x**					
			(93)		**x**			**x**		**x**								**x**				
			(95)							**x**							**x**					
			(12)						**x**	**x**		**x**		**x**	**x**	**x**	**x**	**x**	**x**			**x**
			(47)	**x**	**x**	**x**	**x**	**x**				**x**								**x**	**x**	
			(60)	**x**	**x**					**x**				**x**		**x**			**x**			
			(72)	**x**						**x**					**x**							
			(48)			**x**				**x**				**x**		**x**						**x**
	Canada		(66)	**x**	**x**	**x**							**x**				**x**					

Region			Source	Framework										Established Essential Services								
	Country	State/ Local		I.O.M. Three Core Functions of Public Health (U.S.A.)																		
			(12,38,42, 47,48,50)	Assessment		Policy Development			Assurance													
			(40,42,44, 48,51–56)	10 Essential Public Health Services (U.S.A.)																		
				Health Assessment and Monitoring	Investigation/Surveillance	Inform/educate/Health Promotion	Partner Engagement & Advocacy	Policy Development & Planning	Regulation/ Enforcement	Link & provide health services	Workforce strengthening	Evaluation of health services	Research	Communicable Disease Control	Chronic Disease & Injury Prevention	Health Protection	Emergency Planning & Response	Laboratory Services	Licensing	Program Implementation	Resource & Organization Mgmt.	Vital Statistics
North America																						
	Canada		(2,4,32,63, 96)	x	x	x									x	x						
			(97)	x		x				x			x	x	x	x	x					
			(98)	x	x				x					x	x	x	x					
			(75)		x	x									x	x						
			(73)			x								x	x	x	x					
		Ontario	(61)	x	x	x			x	x			x	x	x	x	x				x	
			(34)			x				x				x	x	x	x					
			(77)	x	x	x		x		x					x	x	x					
		British Colombia	(53)	x	x	x						x			x	x	x					
		Alberta	(61)				x		x	x				x			x				x	

Region			Source	Framework																		
	Country	State/ Local		I.O.M. Three Core Functions of Public Health (U.S.A.)										Established Essential Services								
			(12,38,42,47,48, 50)	Assessment		Policy Development			Assurance													
			(40,42,44,48,51–56)	10 Essential Public Health Services (U.S.A.)																		
				Health Assessment and Monitoring	Investigation/Surveillance	Inform/educate/Health Promotion	Partner Engagement & Advocacy	Policy Development & Planning	Regulation/ Enforcement	Link & provide health services	Workforce strengthening	Evaluation of health services	Research	Communicable Disease Control	Chronic Disease & Injury Prevention	Health Protection	Emergency Planning & Response	Laboratory Services	Licensing	Program Implementation	Resource & Organization Mgmt.	Vital Statistics
North America																						
	Canada	Newfoundland and Labrador	(61)		x	x			x	x			x	x	x		x				x	
		New Brunswick	(61)		x				x	x				x		x	x				x	
		Manitoba	(61)		x	x	x		x	x			x	x	x	x	x				x	
		Saskatchewan	(61)	x	x	x	x		x	x			x	x	x	x	x				x	
South America																						
	Latin American Region		(53)	x	x	x	x	x	x		x	x	x				x				x	

Region			Source	Framework																		
	Country	State/ Local		I.O.M. Three Core Functions of Public Health (U.S.A.)										Established Essential Services								
			(12,38,42, 47,48,50)	Assessment		Policy Development			Assurance													
			(40,42,44, 48,51–56)	10 Essential Public Health Services (U.S.A.)																		
				Health Assessment and Monitoring	Investigation/Surveillance	Inform/educate/Health Promotion	Partner Engagement & Advocacy	Policy Development & Planning	Regulation/ Enforcement	Link & provide health services	Workforce strengthening	Evaluation of health services	Research	Communicable Disease Control	Chronic Disease & Injury Prevention	Health Protection	Emergency Planning & Response	Laboratory Services	Licensing	Program Implementation	Resource & Organization Mgmt.	Vital Statistics
Asia																						
	India		(53,64)	x	x	x		x	x	x	x	x	x	x			x				x	
Middle East																						
	Israel		(48,53)	x			x	x	x		x	x	x		x	x	x					
Pacific																						
	Fiji, Malaysia, Viet Nam		(53,58)	x		x		x	x		x	x	x		x						x	
	Australia		(53)	x	x	x	x	x		x				x	x	x					x	
	New Zealand		(53)	x	x	x				x		x				x						
Europe																						
	European Region		(53)	x		x	x	x		x			x	x		x	x	x				

Region			Source	Framework										Established Essential Services								
	Country	State/ Local		I.O.M. Three Core Functions of Public Health (U.S.A.)																		
			(12,38,42, 47,48,50)	Assessment		Policy Development			Assurance													
			(40,42,44, 48,51–56)	10 Essential Public Health Services (U.S.A.)																		
				Health Assessment and Monitoring	Investigation/Surveillance	Inform/educate/Health Promotion	Partner Engagement & Advocacy	Policy Development & Planning	Regulation/ Enforcement	Link & provide health services	Workforce strengthening	Evaluation of health services	Research	Communicable Disease Control	Chronic Disease & Injury Prevention	Health Protection	Emergency Planning & Response	Laboratory Services	Licensing	Program Implementation	Resource & Organization Mgmt.	Vital Statistics
Europe	European Region		(53)	**x**		**x**	**x**				**x**		**x**		**x**	**x**	**x**				**x**	
	European Region		(53)			**x**	**x**				**x**										**x**	
	Eastern Europe		(35)	**x**		**x**				**x**				**x**	**x**	**x**						
	Eastern Mediterranean		(36)		**x**	**x**									**x**	**x**	**x**					
	Eastern Mediterranean		(53)		**x**	**x**	**x**	**x**			**x**		**x**			**x**	**x**			**x**	**x**	
	U.K.		(83)	**x**	**x**	**x**			**x**	**x**		**x**	**x**	**x**	**x**		**x**					
			(53)	**x**	**x**		**x**		**x**		**x**	**x**	**x**							**x**		
Global			(37)		**x**	**x**			**x**							**x**	**x**					
			(33)	**x**		**x**									**x**	**x**				**x**		
**Total**			**39**	**24**	**25**	**30**	**13**	**11**	**16**	**22**	**9**	**11**	**16**	**18**	**23**	**26**	**25**	**3**	**2**	**4**	**14**	**2**

Additional References

93. Benton K, Polite S. The Disconnect between Public Health and Health Care. Health Prog. 2016;97(2):58–61.

94. Halverson PK. Embracing the strength of the public health system: why strong government public health agencies are vitally necessary but insufficient. J Public Health Manag Pract. 2002;8(1):98–100.

95. Salinsky E, Gursky EA. The case for transforming governmental public health. Health Aff (Millwood). 2006;25(4):1017–28.

96. Chambers LW, Sullivan SM. Reflections on Canada's public health enterprise in the 21st century. Healthc Pap. 2007;7(3):22–30.

97. Deber R, McDougall C, Wilson K. Public health through a different lens. Healthc Pap. 2007;7(3):66–71.

98. The Chief Public Health Officer's report on the state of public health in Canada, 2008. [Internet]. The Chief Public Health Officer's report on the state of public health in Canada, 2008. 2008. Available from: http://www.phac-aspc.gc.ca/cpho-acsp/index-eng.php

99. Mays GP, McHugh MC, Shim K. Institutional and Economic Determinants of Public Health System Performance. Am J Public Health. 2006;96(3):523–31.
