# Supplementary material for: Defining and classifying public health systems: a critical interpretive synthesis
Source: Health Res Policy Syst. 2020 Jun 16;18:68. doi: 10.1186/s12961-020-00583-z (PMC7296190; doi:10.1186/s12961-020-00583-z)
Supplement: Supplementary file 5 — Additional file 5. Aligning public health systems into the health system arrangements framework. This additional file includes a summary of the coded data sources used to align public health within the health system arrangements framework. [file 12961_2020_583_MOESM5_ESM.pdf]

Appendix 5: Aligning public health systems into the health system arrangements framework

**Public health system arrangements**		
**A. Governance Arrangements**		
	**Public Health Systems**	**Sources discussing arrangements**
**Policy Authority**	• Four levels of policy authority in public health (e.g., international, national, regional, and local.)	(4,39,44,63)
	• Federal governments involved in regulatory functions	(4,32,39,48,54,57,61–64)
	• Policy authority de-centralized (e.g., state/provincial/territory/municipality level)	(4,12,32,34,37,44,46,49,57–60)
	• Legislation mandates performance and other procedures	(44,49,58,60,71,82,95)
	• Defined powers of governmental public health agencies vary.	(99)
	• Leadership, expertise and guidance, advocacy (e.g., governmental public health agencies) (e.g., chief public health officer assumes authority over minister of health on issues of public health) (e.g., senior public health managers)<br>• Resource stewardship and oversight (4,73,82)	(4,34,54,58,64,72,95,96)
	• Political and financial influence and support can persuade agencies to target specific public health objectives	(64)
	• In national public health emergencies, the federal government leads and communicates with foreign governments, and health agencies	(4,39)
**Organizational authority**	• Boards of health (e.g., state, local) (e.g., independent, elected, appointed) (e.g., public health professionals, citizens, consumers, educators, and business professionals)	(34,44,57,60,61,63,65,68,80,82)
	• Who can approve health department budgets, adopt regulations, set and impose fees	(4,32,44,57,60–63,68,71)
**Commercial authority**	N/A	N/A

**Professional authority**	• Professionals are represented and regulated by their associated regulatory colleges	(57)
**Consumer and stakeholder involvement**	• citizens provide informed consent when participating in public health clinical services	(57)
	• Stakeholder organizations are given a voice in policy and organizational decisions<br>(e.g., private citizens and consumers)<br>(e.g., communities, boards of health)<br>• Advocacy groups influence policy	(44,57,58,63,68,80)
**Partnership Engagement**	• Partnerships and collaboration occurs at all levels of government, and to varying degrees of collaboration between other public, private and community organizations<br>• Building and maintenance of services through coordination is often mandated<br>• Dependency on partners to deliver and contribute to programs.	(32,39,44,46,54,56,57,62,64,71)
**B. Delivery Arrangements**		
	**Public Health Systems**	**Sources discussing arrangements**
**How care is designed to meet consumers' need**	• Public health functions carried out by all levels of government, federal, state/provincial, local, but most activities are carried out at state/provincial level or locally<br>(e.g., protection or promotion marketing is more effective from the federal level)<br>(e.g., programs designed at state/provincial and local levels)<br>(e.g., immunizations delivered at the local level)	(2,34,38–40,46,48,57–59,62,62,63,65,68–71,96)
	• Size of jurisdictions influence support, human resources	(32,38,46,55,57,59,61,76)
	• Organizational structures	(44,45)
	• Funding to target specific programs and groups	(38,57,72)

**Who care is provided by**	• Care is provided at all levels of government (57)<br>• Care delivered by multiple organizations outside of government (46)<br>• Multidisciplinary nature of public health system means wide range of professionals participate in public health system<br>(e.g., by both regulated and unregulated professionals)	(5,57,63,73,75)
**Where care is provided**	• Delivery of public health services occurs in multiple public and private settings<br>(e.g., schools, homes, offices, clinics, community)	(57,61)
**With what supports care is provided**	• Public health relies on data<br>e.g., public health laboratories, surveillance<br>• Technology (66)<br>(e.g., eHealth, internet, media)	(2,40,51,57,59)
**Partnership**	• Partnerships with other governmental, non-governmental, and community organizations<br>(e.g., emergency response, reporting, surveillances)	(37,40,49,54,62,71,73,74,80)
**C. Financial Arrangements**		
	**Public Health Systems**	**Sources discussing arrangements**
**Financing systems**	• General taxation	(32,39,47,52,57–59,62,69,76)
	• Dedicated funding streams/"ear-marked/targeted funding" from taxes charged on consumer goods, such as fuel or tobacco	(32,46,59,62)
	• Service fees	(44,47,52,59)
	• Private sector funding from non-government organizations, such as non-profit and for-profits and development agencies	(52,58,61)
	• Partnerships/public sector collaborations between different Ministries and other partners	(47,59,65)
	• Intersectoral collaboration between public and private sectors	(39)
	• Public health underfunded<br>• All level of government in the United States funded public health, although spending accounts for 1-3 percent	(5,32,38,39,44,57) (51)
	• Public health received 13.5% of fiscal Department of Health budget to conduct broad range of services (1997)	(72)
	• Cost-shared between governments	(4,52,55,57,62,63,65,69)

**Funding Organizations**	• Informal funding for non-mandatory programs (12)	
	• Allocate funds for specific public health activities<br>• Allocated by funding formulas<br>• High priority programs receive support from external factors<br>• Pay-for-performance arrangements	(38,39,46,47,58,59)
	• Sources of funding vary	(32,38,44,55)
**Remunerating providers**	• Not defined	N/A
**Purchasing products and services**	• Mandatory programs and services are funded	(57,58,65)
	• Funding individual public health services.	(44)
**Incentivizing consumers**	• N/A	N/A

Additional References

93. Benton K, Polite S. The Disconnect between Public Health and Health Care. Health Prog. 2016;97(2):58–61.

94. Halverson PK. Embracing the strength of the public health system: why strong government public health agencies are vitally necessary but insufficient. J Public Health Manag Pract. 2002;8(1):98–100.

95. Salinsky E, Gursky EA. The case for transforming governmental public health. Health Aff (Millwood). 2006;25(4):1017–28.

96. Chambers LW, Sullivan SM. Reflections on Canada's public health enterprise in the 21st century. Healthc Pap. 2007;7(3):22–30.

97. Deber R, McDougall C, Wilson K. Public health through a different lens. Healthc Pap. 2007;7(3):66–71.

98. The Chief Public Health Officer's report on the state of public health in Canada, 2008. [Internet]. The Chief Public Health Officer's report on the state of public health in Canada, 2008. 2008. Available from: http://www.phac-aspc.gc.ca/cpho-acsp/index-eng.php

99. Mays GP, McHugh MC, Shim K. Institutional and Economic Determinants of Public Health System Performance. Am J Public Health. 2006;96(3):523–31.
